# Transgenic mice overexpressing nesfatin/nucleobindin-2 are susceptible to high-fat diet-induced obesity

**DOI:** 10.1038/nutd.2015.42

**Published:** 2016-03-07

**Authors:** H Shimizu, M Tanaka, A Osaki

**Affiliations:** 1 Department of Diabetes and Endocrinology, International University of Health and Welfare (IUHW) Hospital, Nasushiobara, Japan; 2 Center for Medical Science, IUHW, Otawara, Japan; 3 Department of Medicine and Molecular Science, Gunma University Graduate School of Medicine, Maebashi, Japan

## Abstract

**Background:**

Nesfatin/Nucleobindin-2 (Nesf/NUCB2), a precursor of nesfatin-1, an anorexigenic protein, is ubiquitously expressed in peripheral tissues in addition to the hypothalamus. However, the role of intracellular Nesf/NUCB2 has not been established in the periphery.

**Methods:**

Nesf/NUCB2-transgenic (Tg) mice were generated, and chronological changes of body weight and daily food intake were measured in Nesf/NUCB2-Tg mice fed normal laboratory chow or 45% high-fat diet (HFD). In addition, changes of metabolic markers were evaluated in those mice.

**Results:**

No differences were observed in daily food intake and body weight between Nesf/NUCB2-Tg mice (*n*=11) and their non-Tg littermates (*n*=11) fed normal chow. Nesf/NUCB2-Tg mice showed increased mRNA expression of oxytocin and corticotropin-releasing hormone and decreased mRNA expression of cocaine- and amphetamine-related transcript in the hypothalamus. Nesf/NUCB2-Tg mice fed 45% HFD (*n*=6) showed significantly higher increase in body weight than their non-Tg littermates fed the same diet (*n*=8); however, no difference was observed in daily food intake between these two groups. Further, Nesf/NUCB2-Tg mice fed 45% HFD showed a significant increase in the weight of the liver, subcutaneous fat, and brown adipose tissue and decrease in the expression of uncoupling protein-1 in the subcutaneous fat. Blood glucose levels of Nesf/NUCB2-Tg mice fed 45% HFD were not different from those of their non-Tg littermates fed the same diet. Insulin levels of these Tg mice were significantly higher than those of their non-Tg littermates. Histological analysis showed marked fat deposition in the hepatocytes surrounding the hepatic central veins in Nesf/NUCB2-Tg mice fed 45% HFD.

**Conclusions:**

Overexpression of Nesf/NUCB2 did not change food intake, but increased body weight only in Nesf/NUCB2-Tg mice fed HFD. The results of this study indicate that Nesf/NUCB2 was involved in the development of insulin resistance and fat deposition in the liver, independent of the modulation of energy intake.

## Introduction

Nesfatin-1 is an anorexigenic protein found in the paraventricular nucleus of the hypothalamus.^1^ Nesfatin-1 regulates body weight, sympathetic activities, body temperature and hepatic glucose metabolism in the hypothalamus.^2, 3, 4^ Nesfatin/Nucleobindin-2 (Nesf/NUCB2), a precursor of nesfatin-1, is ubiquitously expressed in the peripheral tissues, including the adipose tissue, liver, pancreas and kidney, in addition to the brain.^5, 6, 7, 8^ However, the role of Nesf/NUCB2 in the peripheral tissues is still unknown. Although circulating levels of nesfatin-1 are associated with body mass index,^6^ the source of these circulating levels is not known to date. We have shown that Nesf/NUCB2 expression in the white adipose tissue is regulated by the hypothalamus via the sympathetic nervous system^5^ and that modulation of Nesf/NUCB2 expression in the peripheral tissues may determine the circulating levels of nesfatin-1.^9^ Circulating nesfatin-1, which may be derived from the peripheral tissues, crosses the blood–brain barrier.^10, 11^ In addition, we have shown that peripheral administration of nesfatin-1 decreases food intake in mice.^12^ These data increase the possibility that nesfatin-1 produced in the peripheral tissues mediates nutritional signals from the peripheral tissues such as adipose tissue to the brain.^13^

Nucleobindin, an EF-hand calcium-binding protein, is a Golgi-resident protein that plays a key role in calcium homeostasis in the Golgi network.^14^ Nucleobindin is also present in the endoplasmic reticulum and mitochondria.^15^ Muscles and adipose tissue of patients with type 2 diabetes mellitus show increased expression of Nesf/NUCB2.^16^ I.v. injection of nesfatin-1 significantly reduces blood glucose in hyperglycemic db/db mice in a time-, dose- and insulin-dependent manner.^17^ This suggests that Nesf/NUCB2 regulates intracellular glucose metabolism in the peripheral tissues. However, the nutritional role of Nesf/NUCB2 in the peripheral tissues has not been established *in vivo*. The present study was undertaken to investigate a possible role of Nesf/NUCB2 as a metabolic signal from the peripheral tissue to the brain, and furthermore, to clarify the involvement in the regulation of intracellular metabolism in peripheral tissues by using Nesf/NUCB2-transgenic (Tg) mouse.

## Materials and methods

### Generation of Nesf/NUCB2-Tg mice

Nesf/NUCB2-Tg mice were generated to produce Nesf/NUCB2 by inserting Nesf/NUCB2 complementary DNA (cDNA) into a construct containing CAG promoter. Briefly, a 1.7-kb fragment of the CAG promoter was ligated to a 1263-bp fragment corresponding to position +202 to +1464 of Nucb2 (BC010459) cDNA, including rabbit beta-globin polyadenylation signal. The linearized 3.6-kb S*alI*–Hin*dIII* CAG-Nucb2 construct was injected into pronuclei of fertilized zygotes of C57BL/6 mice. The zygotes were then transferred into pseudopregnant female mice. The resultant offspring were screened for genomic integration by performing PCR of their tail DNA with CAG-*Nucb2*-specific primers (forward, 5′-CCTACAGCTCCTGGGCAACGTGCTGGTT-3′ reverse, 5′-AGAGGGAAAAAGATCTCAGTGGTAT-3′ PCR product size: 14.5 kb). A single line of Tg mice, which showed highest expression of Nesf/NUCB2, was selected from several independent lines generated, and Tg mice were generated by mating F1 heterozygous Tg male mice with wild-type female mice.

The expression of Nesf/NUCB2 was confirmed in the skeletal muscle of all Nesf/NUCB2-Tg mice and non-Tg mice. In addition, it was demonstrated that Nesf/NUCB2 is overexpressed in the samples of whole brain, kidney, liver and pancreas, but not in those of subcutaneous and mesenteric fat pads of Nesf/NUCB2-Tg mice.

#### Experimental protocol

Nesf/NUCB2-Tg mice and their non-Tg littermates were randomly divided into two groups: (1) mice fed normal laboratory chow (*n*=11; Nesf/NUCB2-Tg mice, *n*=11; non-Tg littermates) and (2) mice fed 45% high-fat diet (HFD; Research Diet, Inc., New Brunswick, NJ, USA) (*n*=6; Nesf/NUCB2-Tg mice, *n*=8; non-Tg littermates). No sample size estimation and blinding were done in those experiments.

Chronological changes in the body weight and daily food intake of Nesf/NUCB2-Tg mice and their non-Tg littermates that were fed normal laboratory chow were monitored from 6 to 20 weeks of age. The mice were killed on ad libitum feeding during the light cycle at 20 weeks of age under isoflurane anesthesia (AbbVie, Inc., Tokyo, Japan), and their blood samples were collected. In addition, whole hypothalami and skeletal muscles were dissected and frozen for further analysis.

Chronological changes in the body weight and daily food intake of Nesf/NUCB2-Tg mice and their non-Tg littermates that were fed 45% HFD were monitored for 14 weeks starting from 6 to 20 weeks of age. The mice were killed at the age of 20 weeks, and their blood samples were collected. In addition, the weight of the subcutaneous white adipose tissue (SWAT), mesenteric white adipose tissue, inter-scapular brown adipose tissue (iBAT), liver and skeletal muscle were measured.

The protocol of these experiments was approved by the Animal Research Committee of IUHW (No. D13002).

#### Western blotting

Frozen muscles, SWAT and inter-scapular BAT were homogenized in ice-cold RIPA buffer (25 mM Tris-HCl (pH 7.5), 150 mM NaCl, 1% (w/v) Nonidet P-40 (Sigma-Aldrich, St Louis, MO, USA), 0.5% sodium deoxycholate and 0.1% SDS) containing 1/100 (v/v) Protease Inhibitor Cocktail (Thermo Fisher Scientific, MA, USA) and were incubated with vortexing at 4 °C for 30 min. The homogenates were centrifuged twice at 18 000*g* and 4 °C for 15 min. The supernatants were collected as protein extracts, and concentration of proteins in these extracts was measured using BCA Protein Assay Kit (Thermo Scientific), with BSA as the standard. Protein extracts were mixed with SDS sample buffer containing 2-mercaptoethanol, heated at 40 °C for 10 min and cooled immediately on ice. Aliquots of the protein extracts containing 15 μg of proteins were resolved by SDS–polyacrylamide gel electrophoresis on 5–20% polyacrylamide gels by using ATTO electrophoresis unit (Tokyo, Japan). The separated proteins were transferred onto polyvinylidene fluoride membranes (PALL Corporation, Port Washington, NY, USA), and the membranes were blocked in 0.05% Triton-TBS containing 3% skimmed milk for 30 min at room temperature. The membranes were then incubated overnight with 1 μg ml^−1^ antibodies against NUCB2 (N-terminal) (Sigma-Aldrich), uncoupling protein-1 (UCP-1), UCP-3, glyceraldehyde-3-phosphate dehydrogenase (GAPDH) or β-actin at 4 °C. Excess antibodies were washed with 0.1% TBST, and the membranes were incubated with horseradish peroxidase-conjugated anti-rabbit antibody (20 000 × dilution; Cell Signaling Technology, MA, USA) for 2 h at room temperature. After washing excess antibodies, the membranes were incubated with SuperSignal West Pico Chemiluminescent Substrate (Thermo Scientific) for 5 min at room temperature, and signals were detected by exposing the membranes to X-ray films. After exposure, the membranes were incubated with 62.5 mM Tris-HCl (pH 7), 1% SDS and 100 mM 2-mercaptoethanol at 37 °C for 30 min to strip the previously used antibodies. Next, the membranes were incubated with 0.05 mg ml^−1^ anti-GAPDH antibody (AbD Serotec, Raleigh, NC, USA) and horseradish peroxidase-conjugated anti-mouse antibody to detect GAPDH (loading control).

#### Real-time PCR

Nesf/NUCB2-Tg mice and their non-Tg littermates that were fed normal chow were killed and their hypothalami were isolated. Total RNA was isolated from the whole hypothalamus by using Pure Link RNA Mini Kit (Ambion by Life Technologies, Carlsbad, CA, USA), according to the manufacturer's instructions. Reverse transcription was performed using High Capacity RNA-to-cDNA Kit (Applied Biosystems, Foster City, CA). Quantitative PCR was performed using TaqMan Fast Advanced Master Mix and Step One Plus System with version 2.1 software (Applied Biosystems), according to the manufacturer's instructions. Primers used for quantitative PCR were also obtained from Applied Biosystems. *Gapdh* was used as the internal control. All analyses, including settings for threshold and quantification cycle values were adjusted automatically by using the default settings. Expression of *Nucb2* cDNA was normalized to that of *Gapdh*. All the PCRs were performed in 96-well plates by using 40 cycles of 95 °C for 20 s and 60 °C for 20 s.

#### Histological examination

Mice fed 45% HFD for 14 weeks (age, 20 weeks) and 22 weeks (age, 28 weeks) were killed, and their livers were dissected. The livers were fixed in 10% formalin solution and were embedded in paraffin. The sections obtained were then stained using hematoxylin and eosin or Masson trichrome (Muto Pure Chemicals Co., LTD, Tokyo, Japan), according to the manufacturer's protocol.

#### Biochemical analysis

Serum immune-reactive insulin (IRI) level was measured using a commercially available ELISA kit (Mercodia, Uppsala, Sweden). Serum triglyceride (TG) and non-esterified fatty acid (NEFA) levels were measured using Labo Assay TG, NEFA (Wako Pure Chemical Industries, Ltd., Osaka, Japan). Serum nesfatin-1 level was measured using an ELISA kit (Shibayagi Ltd. Co., Shibukawa, Japan).

Lipids were extracted from the frozen liver samples of mice fed 45% HFD for 22 weeks, and levels of TG, total cholesterol, free cholesterol and phospholipids were measured enzymatically.

#### Statistical analysis

All data are expressed as means±s.d. Prism 4 (GraphPad Software, Inc., La Jolla, CA, USA) for Macintosh OS X was used for the statistical analysis. *P*-values <0.05 were considered statistically significant. Statistical analysis of chronological changes of body weight (expressed as mean) was performed using two-way analysis of variance, with repeated measurements. After all variables were confirmed to be normally distributed, means of two groups were compared using Student's *t*-test.

## Results

### Changes in the body weight and daily food intake of mice fed normal chow

**Figure 1:**
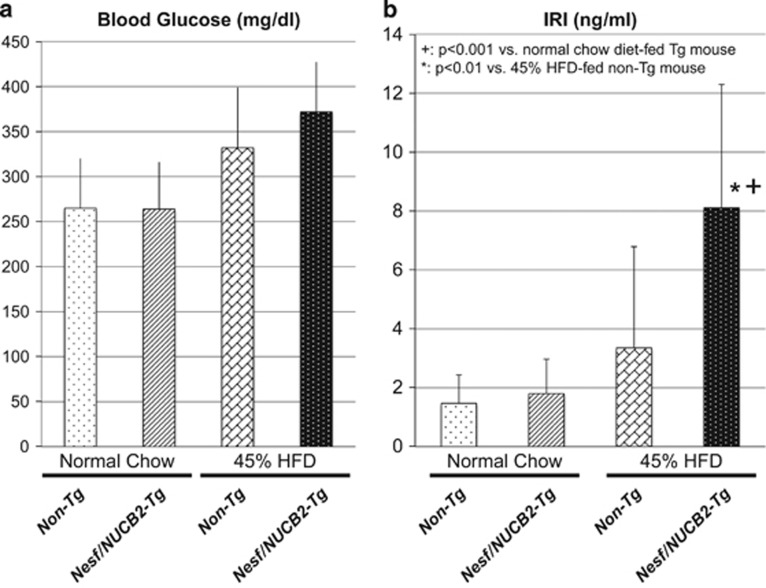
Changes in blood glucose (**a**) and serum immunoreactive insulin (IRI) (**b**) levels in Nesf/NUCB2-Tg mice and their non-Tg littermates fed normal chow and 45% HFD. *n*=11 (Tg mice fed normal chow diet), 11 (non-Tg mice fed normal chow diet), 6 (Tg mice fed 45% HFD) and 8 (non-Tg mice fed 45 HFD).

**Figure 2:**
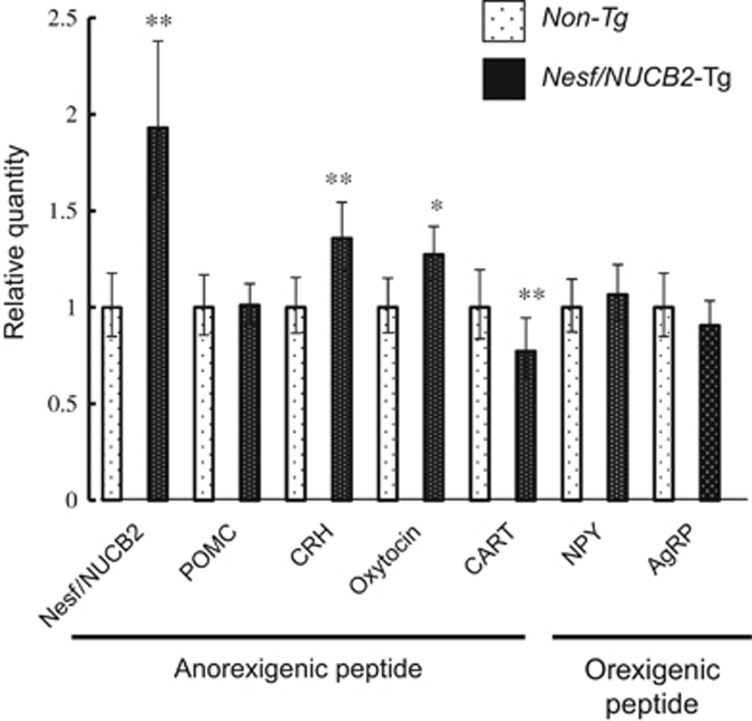
Changes in the mRNA expression of CRH, POMC, NPY, AgRP, CART and nesf/NUCB2 in the hypothalamus of Nesf/NUCB2-Tg mice and their non-Tg littermates fed normal chow. *P<0.05 and **P<0.01 compared with non-Tg mice. *n*=6 (Tg mice), 6 (non-Tg mice).

At 20 weeks of age, no differences were observed in the daily food intake (*P*=0.307) and body weight (*P*=0.825) between Nesf/NUCB2-Tg mice (*n*=11) and their non-Tg littermates (*n*=11) fed normal chow. Moreover, no difference was observed in body weight between Nesf/NUCB2-Tg mice (10.3±1.3 g, *n*=17) and their non-Tg littermates (10.7±1.5 g, *n*=14) after weaning. After sacrifice at 20 weeks of age, no differences were observed in blood glucose and serum IRI levels between Nesf/NUCB2-Tg mice and their non-Tg littermates fed normal chow (Figure 1). Serum nesfatin-1 level was not different between Nesf/NUCB2-Tg mice and their non-Tg littermates at 20 weeks of age (Nesf/NUCB2-Tg mice: 1.22±1.17 ng ml^−1^; non-Tg littermates: 1.07±1.05 ng ml^−1^). Although it was confirmed that the expression of Nesf/NUCB2 was 18.7-times higher in the skeletal muscle of Nesf/NUCB2-Tg mice than the skeletal muscle of their non-Tg littermates. Overexpression of Nesf/NUCB2 in the hypothalamus of Nesf/NUCB2-Tg mice at 20 weeks of age was confirmed using reverse transcription–PCR (Figure 2). The mRNA expression of anorexigenic proteins corticotrophin-releasing hormone (CRH) and oxytocin was significantly increased in the hypothalamus of Nesf/NUCB2-Tg mice. However, the mRNA expression of another anorexigenic cocaine- and amphetamine-related transcript (CART) was significantly decreased while that of proopiomelanocortin was unchanged in the hypothalamus of Nesf/NUCB2-Tg mice. The mRNA expression of orexigenic protein neuropeptide Y (NPY) and agouti-related protein (AgRP) was not significantly changed in the hypothalamus of Nesf/NUCB2-Tg mice and their non-Tg littermates fed normal chow.

### Changes in the body weight and daily food intake of mice fed 45% HFD

**Figure 3:**
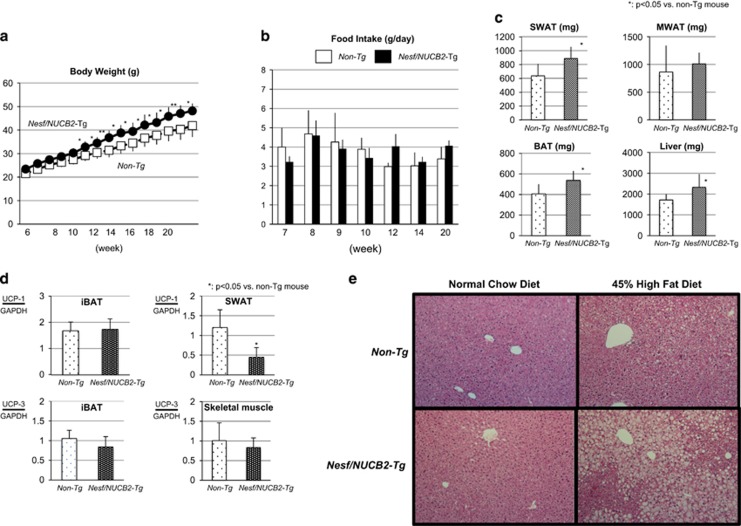
Changes in body weight (**a**) and daily food intake (**b**) between Nesf/NUCB2-Tg mice (*n*=6) and their non-Tg littermates (*n*=8) fed 45% HFD. *P<0.05 and **P<0.01 compared with non-Tg mice. (**c**) Changes in the weight of subcutaneous white adipose tissue (SWAT), mesenteric white adipose tissue (MWAT), inter-scapular brown adipose tissue (iBAT) and liver of Nesf/NUCB2-Tg and non-Tg mice fed 45% HFD. (**d**) Expression of uncoupling protein-1 (UCP-1) and uncoupling protein-3 (UCP-3) in the skeletal muscle, SWAT and iBAT of Nesf/NUCB2-Tg mice and their non-Tg littermates fed 45% HFD. (**e**) Microphotographs of the liver of Nesf/NUCB2-Tg mice and their non-Tg littermates fed normal chow and 45% HFD.

Nesf/NUCB2-Tg mice fed 45% HFD showed significantly greater increase in body weight than their non-Tg littermates fed the same diet; however, no difference was observed in daily food intake between the two groups (Figures 3a and b). Histological analysis after the sacrifice of these mice at the age of 20 weeks showed significant increase in the weight of the SWAT, iBAT and liver in Nesf/NUCB2-Tg mice fed 45% HFD (Figure 3c). UCP-1 expression was significantly reduced in the SWAT of Nesf/NUCB2-Tg mice. However, UCP-1 expression in the iBAT was not different between Nesf/NUCB2-Tg mice and their non-Tg littermates fed 45% HFD (Figure 3d). Moreover, UCP-3 expression in the iBAT and skeletal muscle was not different between nesf/NUCB2-Tg mice and their non-Tg littermates fed 45% HFD.

Although blood glucose levels were not different between Nesf/NUCB2-Tg mice and their non-Tg littermates fed 45% HFD, serum IRI level was significantly higher in Nesf/NUCB2-Tg mice than in their non-Tg littermates (Figure 1), indicating the development of insulin resistance in Nesf/NUCB2-Tg mice fed 45% HFD. No significant difference was observed in the serum levels of TG (Nesf/NUCB2-Tg mice: 176.1±18.7 mg dl^−1^; non-Tg littermates, 167.2±17.5 mg dl^−1^) and NEFA (Nesf/NUCB2-Tg mice: 345.9±55.4 mEq l^−1^; non-Tg littermates: 308.0±66.8 mEq l^−1^) between the two groups. Moreover, increase in serum nesfatin-1 level of Nesf/NUCB2-Tg mice fed 45% HFD (Nesf/NUCB2-Tg mice: 2.18±1.71 ng ml^−1^; non-Tg littermates: 1.46±0.39 ng ml^−1^) was not statistically significant. However, expression of Nesf/NUCB2 was significantly higher in the skeletal muscle of Nesf/NUCB2-Tg mice than in the skeletal muscle of their non-Tg littermates. Histological analysis showed marked fat deposition in the hepatocytes surrounding the hepatic central veins in Nesf/NUCB2-Tg mice fed 45% HFD (Figure 3e), indicating accelerated development of fatty liver.

**Figure 4:**
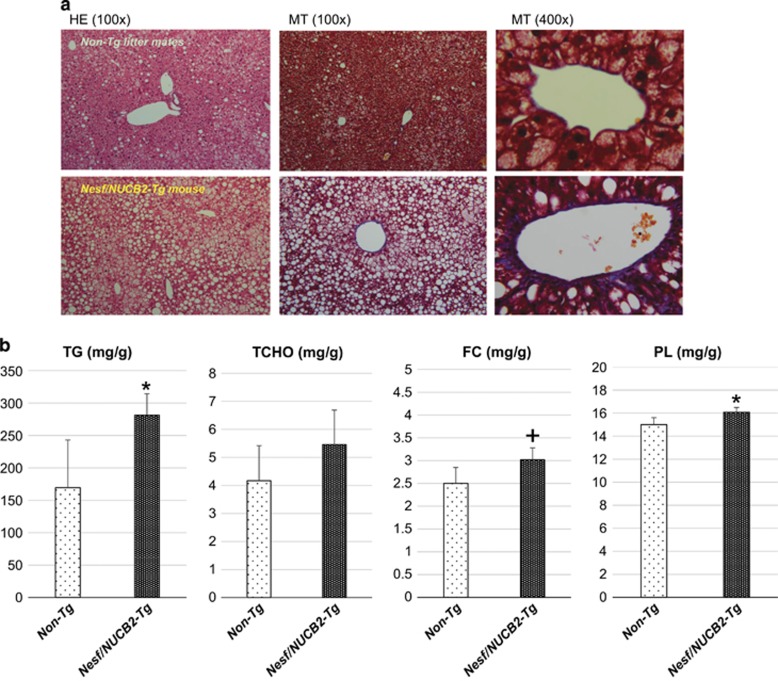
(**a**) Microphotographs of hematoxylin-eosin (HE) and Masson trichrome (MT) staining of the liver of Nesf/NUCB2-Tg mice and their non-Tg littermates fed 45% HFD for 22 weeks. (**b**) Lipid content in the liver. FC, free cholesterol; PL, phospholipid; TCHO, total cholesterol; TG, triglyceride. *P=0.026 and +*P*=0.05 vs non-transgenic littermates. *n*=5 (Tg mice fed 45% HFD), 3 (non-Tg mice fed 45 HFD).

Figure 4a shows the microphotographs of the liver of Nesf/NUCB2-Tg mice and their non-Tg littermates fed 45% HFD for 22 weeks. Fat deposition was visibly higher in the liver of Nesf/NUCB2-Tg mice than in the liver of their non-Tg littermates. Masson trichrome staining showed an increase in collagen fibers around the hepatic central veins. Analysis of the liver lipid content showed that TG level was significantly higher in Nesf/NUCB2-Tg mice than their non-Tg litter mates (Figure 4b).

## Discussion

The present study demonstrated that overexpression of Nesf/NUCB2 failed to show a significant inhibition of body weight from weaning period of Nesf/NUCB2-Tg mice fed a normal chow diet. In addition, daily food intake was not changed in those mice from 7 to 20 week of age. However, the reason why overexpression of Nesf/NUCB2, a precursor protein of anorexigenic molecule, nesfatin-1, failed to show the reduction of food intake and body weight gain could not be clarified in the present studies. The reduction of food intake caused by intracerebroventricularly administered nesfatin-1 is supposed to be mediated by the neurons expressing oxytocin and CRH in the brain.^18, 19^ CRH and oxytocin mRNA were confirmed to be overexpressed in the hypothalamus of Nesf/NUCB2-Tg mice, while the mRNA expression of CART, another anorexigenic protein, was significantly inhibited. Overexpression of hypothalamic CRH and oxytocin mRNA in Nesf/NUCB2-Tg mouse may confirm that nesfatin-1 increases hypothalamic CRH and oxytocin mRNA expressions *in vivo*. In contrast, overexpression of Nesf/NUCB2 inhibited CART mRNA expression in the hypothalamus. Nesf/NUCB2 extensively co-localizes with CART in almost all Nesf/NUCB2-expressing regions of the brain.^20^ We have previously shown that single peripheral administration of the active segment of nesfatin-1 increased CART expression in the nucleus tractus solitarius but not in the arcuate nucleus of the hypothalamus.^12^ The finding that continuous overexpression of Nesf/NUCB2 decreased the mRNA expression of CART in the hypothalamus may be one of possible factors which cancel the anorexigenic effects caused by overexpression of Nesf/NUCB2. No difference in body weight between Nesf/NUCB2-Tg mice and their non-Tg littermates during the weaning period suggested that the effect of Nesf/NUCB2 overexpression in the hypothalamus might be quickly compensated before the weaning period in Nesf/NUCB2-Tg mice. On the other hand, Nesf/NUCB2 co-localizes with NPY in the neurons of the arcuate nucleus of the hypothalamus.^21^ However, mRNA expression of NPY and AgRP was not changed in Nesf/NUCB2-Tg mice, indicating that orexigenic neuropeptides NPY and AgRP are not involved in the regulation of feeding behavior induced by nesfatin-1.

Our previous data that peripheral administration of nesfatin-1 inhibits food intake of mice indicates that an increase of circulating nesfatin-1 in the blood should be important in the inhibition of food intake by nesfatin-1.^12^ However, no increase of circulating nesfaton-1 was not found in Nesf/NUCB2-Tg mice fed normal chow diet and HFD. No increase of circulating nesfatin-1 may, at least in part, explain no changes in feeding behavior of those mice fed both diets. On the other hand, an increase in the body weight of Nesf/NUCB2-Tg mice fed 45% HFD was significantly higher than that of their non-Tg littermates fed the same diet, irrespective of the increase in energy intake. Recent studies have shown that single intracerebroventricular injection of nesfatin-1 increases body temperature immediately^4^ and that cold-activated (Fos-positive) Nesf/NUCB2-expressing neurons in several brain nuclei are involved in cold adaptation, indicating that central nesfatin-1 regulates thermogenesis.^4^ However, continuous peripheral administration of nesfatin-1 reduces cumulative food intake and increases spontaneous physical activity, but does not affect the mRNA expression of UCP-1 in the BAT.^22^ In the present study, expression of UCP-1 was significantly reduced only in the SWAT but not in the BAT of Nesf/NUCB2-Tg mice. Expression of Nesf/NUCB2 in the SWAT of Tg mice was not different from that in the SWAT of their non-Tg littermates. Injection of CART into the paraventricular nucleus of the hypothalamus decreases NPY-induced feeding and induces UCP-1 expression in the white adipose tissue.^23^ Therefore, modulation of UCP-1 expression in the SWAT may be attributed to a neurogenic signal from the brain. In addition, hyperinsulinemia without sustained hyperglycemia triggers diet-induced obesity. Mice that are genetically incapable of developing HFD-induced hyperinsulinemia show increased levels of UCP-1 in the white adipose tissue, but not in the BAT.^24^ Insulin resistance observed in Nesf/NUCB2-Tg mice may be partially attributable to the reduction in UCP-1 expression in the SWAT.

The role of Nesf/NUCB2 as an intracellular signaling molecule is still unknown in peripheral tissues. A study showed that food deprivation increased mRNA expression of NUCB2 in the liver of zebrafish.^25^ A recent study reported that inhibition of central nesfatin-1 activity increased hepatic glucose flux and decreased glucose uptake in the peripheral tissue of rats fed normal chow or HFD.^26^ Although Nesf/NUCB2-Tg mice fed normal chow did not show any changes in metabolic factors, Nesf/NUCB2-Tg mice fed 45% HFD showed significantly higher insulin levels but no changes in blood glucose and serum TG and NEFA levels. Moreover, Nesf/NUCB2-Tg mice fed 45% HFD for 22 weeks showed significant increase in liver weight and TG level, marked fat deposition around the central veins in the liver and increase in collagen fibers. We have confirmed overexpression of Nesf/NUCB2 in the liver of Nesf/NUCB2-Tg mice, although circulating levels of nesfatin-1 were not different between Nesf/NUCB2-Tg mice and their non-Tg littermates. These results indicated that overexpression of Nesf/NUCB2 in the liver might result in insulin resistance associated with excessive accumulation of TG in the liver, in addition to an increase of SWAT weight.

In conclusion, overexpression of Nesf/NUCB2 failed to decrease body weight in Nesf/NUCB2-Tg mice fed normal chow and 45% HFD. Moreover, Nesf/NUCB2-Tg mice fed 45% HFD showed increased accumulation of TG in the liver and insulin resistance. Taken together, these data indicated that Nesf/NUCB2 was involved in the development of insulin resistance and fat deposition in the liver after HFD feeding and that these effects were independent of the modulation of energy intake.
